# Evaluating a Shared Decision Support Tool for Pediatric Cardiopulmonary Arrest: Mixed Methods Usability Study

**DOI:** 10.2196/78736

**Published:** 2026-04-28

**Authors:** Ana Rajic, Sharleen Kayne Olanka, Marco Generelli, Jennifer Davidson, Yiqun Lin, Ryan Kang, Kangsoo Kim, Pierre-Louis Rebours, Marc Ibrahim, Donovan Duncan, Sergio Manzano, Adam Cheng, Alexandre De Masi, Johan N Siebert, Frederic Ehrler

**Affiliations:** 1 Educational Technologies and Learning Sciences (TECFA), Faculty of Psychology and Educational Sciences, University of Geneva, Geneva, Switzerland; 2 Department of Pediatric Emergency Medicine, Geneva Children's Hospital, Geneva University Hospitals, 47 Avenue de la Roseraie, Geneva, 1211, Switzerland, 41 0 22 372 40 72; 3 Departments of Pediatrics and Emergency Medicine, Cumming School of Medicine, University of Calgary, Calgary, AB, Canada; 4 KidSIM-ASPIRE Simulation Research Program, Alberta Children's Hospital, University of Calgary, Calgary, AB, Canada; 5 Department of Electrical and Software Engineering, Schulich School of Engineering, University of Calgary, Calgary, AB, Canada; 6 Division of Computer Sciences, Diagnostic Department, Geneva University Hospitals, Geneva, Switzerland; 7 Pediatric Intensive Care Unit, Alberta Children's Hospital, University of Calgary, Calgary, AB, Canada; 8 Faculty of Medicine, University of Geneva, Geneva, Switzerland

**Keywords:** cardiopulmonary resuscitation, pediatric emergency medicine, decision support systems, clinical, digital health, user-computer interface, health information technology, medical informatics applications, usability testing, simulation training

## Abstract

**Background:**

Effective team communication is critical in pediatric cardiopulmonary arrest management, where delays or miscommunication can jeopardize survival. TeamScreen, a web-based interface displayed on a large screen, was developed to enhance cardiopulmonary resuscitation (CPR) by providing real-time visualization of clinical data and resuscitation steps aligned with the American Heart Association pediatric advanced life support algorithms.

**Objective:**

This study evaluated the usability of the TeamScreen Figma prototype, evaluating how efficiently and accurately experienced emergency physicians and nurses retrieved critical information during a simulated pediatric in-hospital cardiac arrest scenario. Although no strict time constraints were imposed, participants were instructed to perform the tasks as spontaneously and as quickly as possible.

**Methods:**

Usability testing involved 20 pediatric emergency physicians and nurses with varied CPR experience. Participants performed 21 information retrieval tasks within a simulated pediatric cardiac arrest scenario (shockable rhythm). The data collected included audio-video recordings via the think-aloud method and participant responses to the Post-Study System Usability Questionnaire (PSSUQ) version 3 and a posttest survey. Effectiveness, efficiency, and satisfaction were measured by task completion rates, time-on-task metrics, and PSSUQ scores, respectively. Think-aloud data were analyzed for usability issues using Nielsen Norman Group’s rating scale and Bastien and Scapin’s ergonomic criteria.

**Results:**

Five physicians and 15 nurses achieved a mean task success rate of 81.19% (SD 16.87%), with a mean completion time of 8.13 (SD 7.07) seconds, calculated across all 21 tasks and all participants. PSSUQ scores reflected high satisfaction (mean 2.40 [SD 1.24] of 7.00; the lower the better), notably for information clarity and system utility. Qualitative analyses identified 16 usability issues, of which 5 were deemed major, primarily involving information visibility, navigation, and density, highlighting areas for interface and workflow enhancement.

**Conclusions:**

The usability evaluation confirmed TeamScreen’s potential to improve real-time information access during pediatric CPR, with high task success and satisfaction scores supporting its role in aiding decision-making. Challenges with visibility, navigation, and information density require further refinement. These findings will guide improvements and inform the design of multicenter trials to assess TeamScreen’s efficacy in simulation-based resuscitation settings.

## Introduction

### Context and Problem Statement

In-hospital pediatric cardiac arrests (IHCA), although rare, require immediate, coordinated response from resuscitation teams under intense pressure to provide guideline-compliant clinical care [1]. Standardized protocols, such as the American Heart Association pediatric advanced life support (PALS) guidelines, emphasize uniform responses [2], yet adherence remains suboptimal, with frequent deviations from recommended timing of defibrillation and drug administration, errors in dosing, and omissions of key steps in both real and simulated pediatric cardiac arrest scenarios [3-6]. These deviations are strongly influenced by human factors such as high cognitive load, stress, impaired decision-making, communication breakdowns, and challenges in maintaining team situational awareness and a shared mental model during complex emergencies [4]. These lapses compromise quality of care and survival rates [7]. As a result, there is increasing interest in tools that support team cognition and real-time guideline adherence during pediatric cardiopulmonary resuscitation (CPR).

Digital cognitive aids and decision support systems have emerged as promising approaches to mitigate these problems. In adult and mixed emergency settings, cognitive aids and electronic decision support tools have been associated with fewer errors and better adherence to resuscitation algorithms in simulated IHCA [8-11]. In pediatrics, few are purpose-built for real-time use in resuscitation [12]. Tablet-based apps were developed specifically to support pediatric cardiac arrest management and showed good usability in high-fidelity simulation with reduced deviations from guideline recommendations [13-15]. A recent systematic review of cognitive aids in resuscitation similarly concluded that cognitive aids tend to reduce deviations from guidelines and improve resuscitation performance in simulated settings across adult and pediatric populations [16]. However, most existing systems are designed as single-user, handheld interfaces that primarily support the team leader, rather than functioning as shared, team-centered displays that make time-critical information visible to the entire resuscitation team in real time.

Only a few technological solutions have been conceived as large situation displays to enhance team communication and situational awareness during emergency resuscitations. Pilot work in emergency departments has suggested that dedicated situation displays can facilitate teamwork, communication, and perceived situational awareness among resuscitation teams [17,18]. More recently, an interactive, large-screen clinical decision display designed for team-wide viewing has been evaluated and shown to improve adherence to guideline time intervals in simulated advanced cardiac life support [8]. Despite these advances, pediatric-specific evidence for team-centered digital decision support during IHCA remains limited, and there is a need for tools explicitly designed around the information needs and workflows of pediatric resuscitation teams.

**Figure 1.**
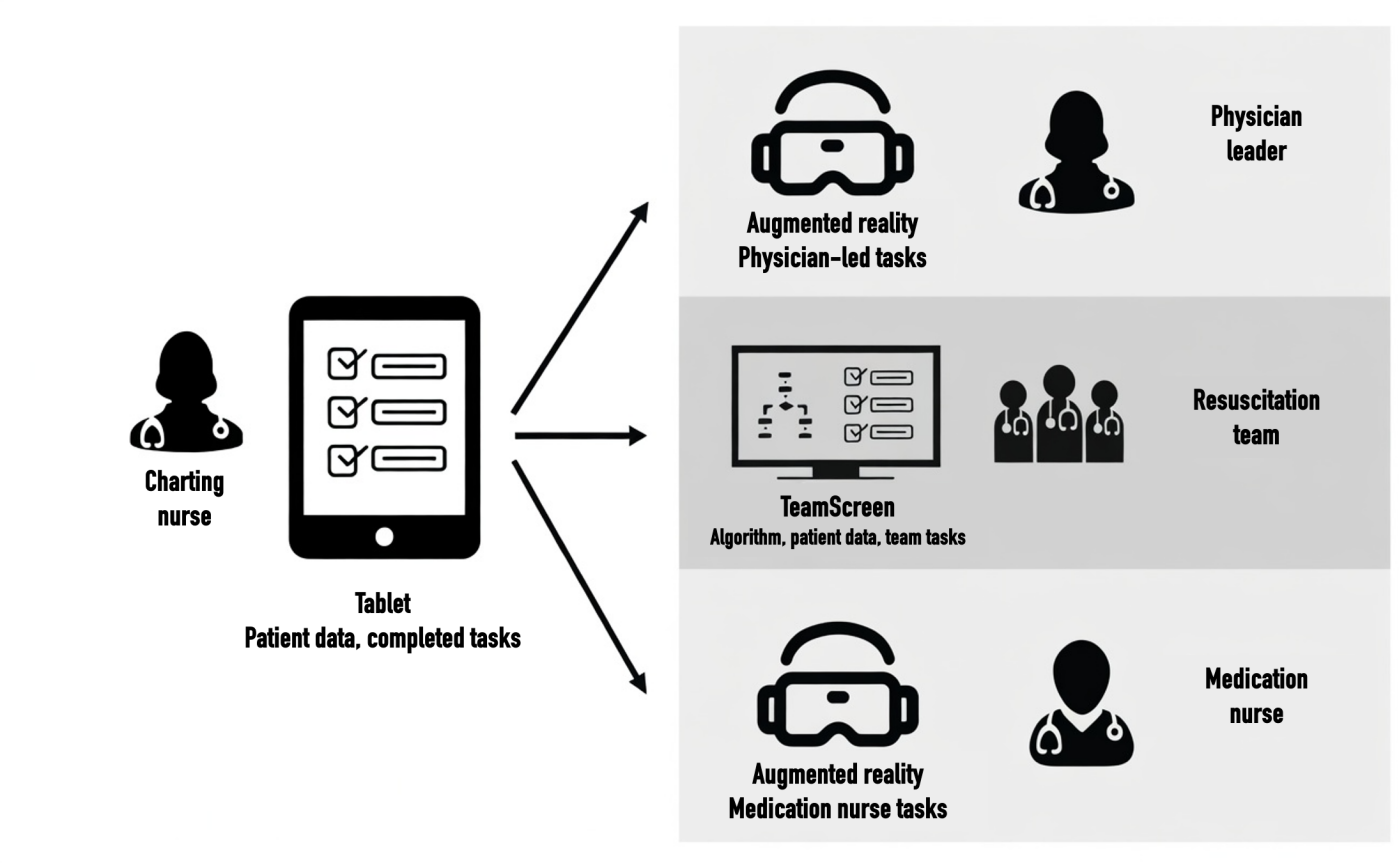
Components of Interconnected and Focused Mobile Applications on Patient Care Environment–augmented reality (InterFACE-AR). The system integrates multiple digital tools to support pediatric resuscitation teams. The Guiding Pad (tablet), used by the charting nurse, captures patient data and completed tasks, distributing real-time information to role-specific interfaces. The TeamScreen (web-based app displayed on a large screen) provides a centralized overview of the resuscitation algorithm, patient data, and team tasks for the entire resuscitation team. Augmented reality (AR) headsets (HoloLens 2) deliver targeted, real-time guidance to the physician leader (for physician-led tasks) and the medication nurse (for medication preparation and administration).

To address these limitations, we developed Interconnected and Focused Mobile Applications on Patient Care Environment (InterFACE), a suite of interconnected digital tools (Figure 1) designed to enhance team coordination and PALS adherence during pediatric CPR [19,20]. Building on an earlier prototype [15] that demonstrated improvements in defibrillation timing and adherence to resuscitation algorithms but also highlighted risks of cognitive overload, we refined the system into an updated concept. This system integrates role-specific, interconnected digital tools—TeamScreen, Guiding Pad (a tablet app), and augmented reality (AR) headsets [21]—to standardize care, reduce variability, and enhance CPR quality [22]. TeamScreen provides real-time resuscitation oversight, with shared access to drug and shock dose calculations and PALS workflows to support the entire team [20]. Developed iteratively with multidisciplinary input from 2 pediatric emergency departments, it addresses prior shortcomings and aligns with calls for innovative resuscitation aids [16].

The design and evaluation of InterFACE-AR followed human factors and usability engineering recommendations for high-risk medical technologies. Regulatory guidance from the US Food and Drug Administration on applying human factors and usability engineering to medical devices emphasizes structured processes, identifying critical tasks, conducting formative evaluations, and performing summative usability testing to minimize use-related risks [23]. Usability research in health information technology further supports the use of task-based testing and think-aloud methods as core, user-centered approaches to uncover interaction problems in complex interfaces [24,25]. In this context, this study focuses specifically on evaluating the usability of the updated TeamScreen interface during a simulated pediatric IHCA scenario, as an essential step before broader assessments of its impact on team performance and clinical outcomes.

### Objective

This study aimed to evaluate the usability of the TeamScreen app in supporting resuscitation teams during pediatric IHCA management. By assessing the prototype’s strengths and limitations, we sought to identify potential usability challenges and propose targeted improvements. The insights gathered were intended to inform the refinement of TeamScreen ahead of its full-scale clinical deployment and to contribute to the broader development of digital tools aimed at enhancing team performance and adherence to resuscitation guidelines.

## Methods

### Study Design

The usability of the TeamScreen app was assessed through a scenario-based information retrieval evaluation, combining both quantitative and qualitative methods. Multitask quantitative and qualitative usability metrics were used and are described in detail in subsequent sections. Usability was defined, according to ISO 9241‐210, as “the extent to which a product can be used by specified users to achieve specified goals with effectiveness, efficiency, and satisfaction in a specified context of use” [26]. The usability of a digital tool can be assessed by how effectively and completely users are able to perform key tasks aligned with its core functionalities. In contrast, poor usability often results in reduced task efficiency, incomplete goal achievement, or even abandonment of the technology [27]. Effectiveness was measured by task completion rates. Efficiency was evaluated through time-on-task metrics. User satisfaction was assessed using the Post-Study System Usability Questionnaire (PSSUQ) version 3 [28] and a custom posttest evaluation questionnaire. In addition, think-aloud discussions were recorded verbatim and analyzed to extract key user insights and perceptions throughout the sessions.

### TeamScreen App Overview: A Core Component of InterFACE-AR

TeamScreen is a web-based app designed to centralize and display, in real time, critical information related to the patient’s condition and resuscitation progress to support adherence to PALS algorithms during CPR. Displayed on a 75-inch wall-mounted screen in the resuscitation room, it provides resuscitation teams with a clear, shared overview of clinical status and required actions, aligned with PALS algorithms. This dynamic interface offers a visual overview of key data such as patient information (eg, weight, pulse presence, cardiac rhythm, and ventilation status), intervention timelines, upcoming clinical tasks, and medication dosing details without requiring direct user interaction. Instead, it relies on input from the charting nurse via the Guiding Pad, a tablet app that dynamically records interventions tailored to the patient’s status. TeamScreen is part of the broader InterFACE-AR suite [22], named for its AR foundation, which also includes AR headsets (HoloLens 2, Microsoft Corp), worn by the team leader and medication nurse [21]. These deliver role-specific guidance via mixed reality, an advanced form of AR that—unlike standard overlays—enables interactive 3D holograms anchored in the environment [29], to support clinical decision-making and medication preparation. The usability testing approach mirrored this role distribution, reflecting real-world dynamics where the Guiding Pad feeds data to TeamScreen for team-wide visibility. These tools collectively aim to improve situational awareness, communication, and adherence to PALS guidelines during IHCA, ensuring more efficient and guideline-compliant interventions. The TeamScreen prototype used in this study replicated all core functionalities of the current InterFACE-AR TeamScreen module, including the algorithm flow, task lists, timers, and weight-based drug and shock displays, but it was operated as a stand-alone simulated interface that was not technically connected to the AR headsets or live clinical systems. The Guiding Pad and AR headset components will be described and evaluated in separate studies.

### Participants and Setting

#### Inclusion Criteria and Recruitment

The inclusion criteria comprised physicians and nurses working in the Pediatric Emergency Department of the Geneva University Hospitals in Switzerland; fluent in French at a C1 level; and with prior experience in pediatric resuscitation, gained through real clinical practice, simulation training, or both. Individuals involved in the development of the InterFACE project were excluded from the study to avoid bias.

Sample size was established at 20 participants. This number is consistent with established guidelines for usability testing in high-risk, domain-specific systems, where 15 to 20 participants are generally considered sufficient to identify the majority of critical usability issues. This recommendation is supported by prior work in the field, particularly in the context of summative evaluations of medical technologies [30]. The evaluation framework focused on interactions between the user, the task, and the system, intentionally excluding the user’s actual clinical environment from the assessment [31]. Recruitment was carried out by 2 senior attending physicians in pediatric emergency medicine (SM and JNS).

#### Ethical Considerations

The study received ethical approval from the Geneva Cantonal Research Ethics Committee (reference Req-2023‐00162). All participants received an information sheet detailing the study’s objectives and provided written informed consent before participating. Participants could opt out at any time without providing a reason and without any consequences. Participants received no compensation for their participation. All data were anonymized to ensure confidentiality. Explicit consent was obtained from participants to use their image for publication.

#### Test Setting

**Figure 2.**
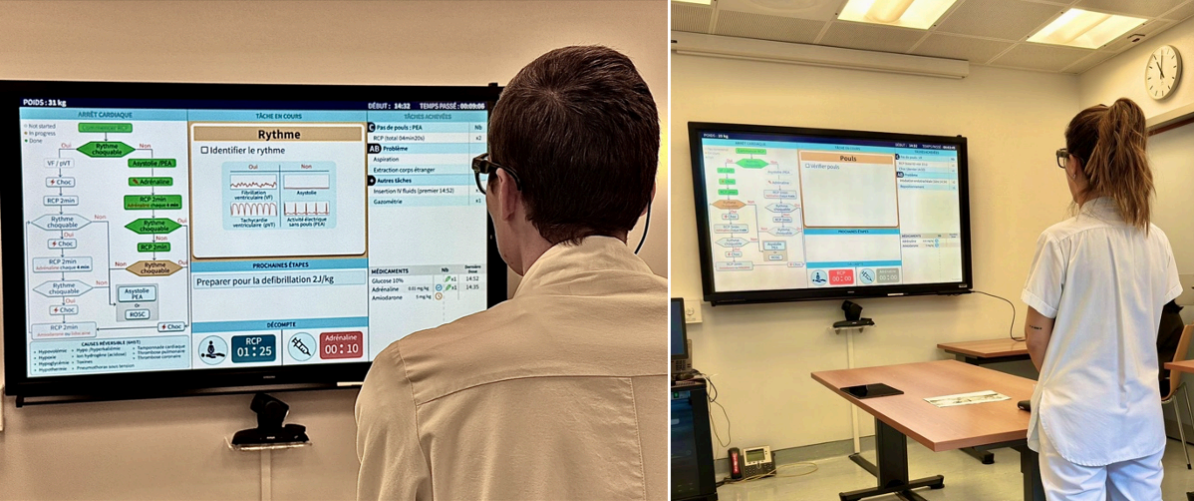
Usability test setting with TeamScreen. Two health care professionals interact with TeamScreen, displayed on a 75-inch wall-mounted screen, during the usability testing session. The participants stand at a standardized distance while engaging in scenario-based tasks to assess system usability. The interface provides real-time guidance on pediatric advanced life support resuscitation algorithms. The test environment simulates a clinical setting, with technical equipment used for data collection and analysis.

The usability evaluation was conducted in a conference room, equipped with a prototype of the TeamScreen system displayed on a 75-inch wall-mounted Samsung Neo QLED screen (model QE75QN90). This standardized setup was designed to simulate real clinical environment and enable technical data collection. Participants stood 2.7 meters from the screen, with a table placed in front of it to simulate a resuscitation bed (Figure 2). This conference room layout (screen position, distance, and table simulating the resuscitation bed) was chosen to approximate a pediatric emergency department resuscitation bay, with the TeamScreen acting as the central “wall monitor” around which the simulated scenario unfolded. Testing sessions were conducted by 3 examiners (ADM, SO, and AR), each responsible for different aspects of the evaluation. SO operated the Guiding Pad connected to the TeamScreen and acted as the charting nurse, simulating real-time input of clinical actions to drive the system forward in alignment with the usability tasks presented to participants. AR facilitated the sessions, administered instructions, and guided the participants through the PALS algorithm, questionnaires, and posttest survey. All verbal responses and think-aloud reflections were recorded using lapel microphones. Data collection tools included a demographic questionnaire, the PSSUQ version 3, and a custom posttest survey, all administered digitally via Microsoft Forms, with responses stored in real time.

### Test Procedure

#### Procedure

**Figure 3.**
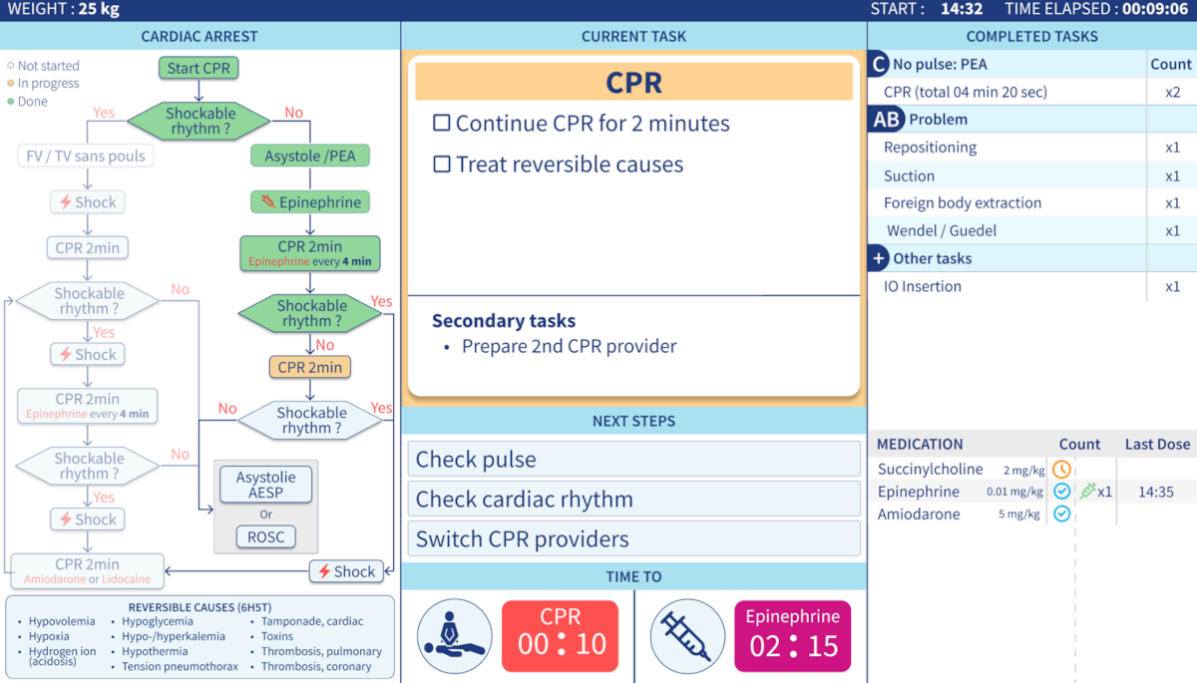
TeamScreen prototype display during a simulated pediatric in-hospital cardiac arrest, showing the cardiopulmonary resuscitation (CPR) algorithm (left) and the current and upcoming tasks (center), with timer boxes for CPR cycles and epinephrine administration displayed at the bottom. The right-hand panel displays completed tasks and the medication list with weight-based dosing. Succinylcholine is included as one of several selectable intubation drugs to test navigation and selection within the interface and should not be interpreted as a recommendation for its use during ongoing cardiac arrest.

Each session involved the same screen-based simulated pediatric resuscitation scenario, featuring a child aged 8 years (25 kg) who had sustained an electrification injury and subsequently developed a witnessed IHCA in an emergency department resuscitation bay. The initial rhythm was ventricular fibrillation/pulseless ventricular tachycardia, and the scenario was structured to follow the typical progression of a case of shockable IHCA requiring high-quality CPR, repeated rhythm checks, defibrillation, and drug administration. No manikin was used, and participants were not required to perform physical life-saving interventions; the focus was exclusively on interacting with the TeamScreen. Using TeamScreen as the shared “wall display” for the resuscitation, participants were required to complete a series of information retrieval tasks, covering key aspects of CPR resuscitation such as pulse assessment (no pulse), defibrillation, medication management (epinephrine, amiodarone, and succinylcholine as an intubation drug option), and cardiac rhythm identification (ventricular fibrillation/pulseless ventricular tachycardia). The scenario included several critical steps that had to be completed before return of spontaneous circulation (ROSC) could be achieved, such as recognizing the absence of a pulse, initiating chest compressions, performing defibrillation, and delivering the appropriate medications at the correct timing. The objective was to evaluate participants’ ability to locate and interpret critical information displayed on the TeamScreen interface without prior training. The usability testing followed a standardized protocol:

1. Participant onboarding: upon arrival, participants were provided with an information sheet, and they signed an informed consent form. The study’s purpose and procedure were explained, after which the participants completed a demographic questionnaire assessing their experience and expertise in pediatric resuscitation.
2. Equipment setup: participants were fitted with Tobii Pro Glasses 3 (Tobii AB) for eye-tracking analysis, and lapel microphones were used to record verbal feedback throughout the session.
3. Familiarization: clinicians who had been involved in the conception or technical development of the InterFACE-AR system were excluded. The remaining participants had not previously used the TeamScreen prototype and were exposed to it only through a short, standardized briefing immediately before the usability session. They were briefly presented with a static TeamScreen prototype (Figure 3) via FIGMA (Figma Inc) and asked to describe their observations. This phase served to ease their interaction with the interface before engaging in task execution.
4. Scenario execution and task completion: participants proceeded with the simulated IHCA scenario, performing 21 sequential information retrieval tasks (Table 1) using the think-aloud method [32]. Each task corresponded to a TeamScreen display, with additional information progressively introduced as the session advanced. These tasks were designed to assess comprehension and eye navigation on the interface. Participants were asked to verbalize their thought processes while retrieving information, guided by 1 examiner (AR), who intervened only if a participant struggled to reach a final answer. Task success, completion time, and time required for task presentation were recorded.
5. Posttest debriefing: upon completion, participants filled out the PSSUQ version 3; the responses were used to assess their satisfaction with TeamScreen. A semistructured interview was conducted to gather qualitative feedback regarding usability, strengths, and areas for improvement.
6. Closing and follow-up: participants were thanked for their time, informed about the next steps of the project, and invited to share any additional comments or suggestions.

#### Scenario and Tasks

The IHCA scenario was standardized and tailored to each participant’s professional role. Pediatric emergency physicians were assigned the role of team leader, and nurses were assigned the role of medication nurse. The scenario was co-developed by 2 pediatric emergency physicians (SM and JNS) and designed in parallel with 21 information retrieval tasks (Table 1) predefined by 3 examiners (ADM, SO, and AR), ensuring alignment between task execution and the corresponding TeamScreen displays.

Within the broader InterFACE-AR concept, the complete system is intended to provide individualized, role-specific decision support to the team leader and medication nurse through optically see-through AR headsets and to display a dynamic road map for patient care on TeamScreen, both components being coordinated via the Guiding Pad tablet app. This human factors study focuses solely on the usability of the TeamScreen app across a series of information retrieval tasks (Table 1). In the full system, weight-based medication doses are automatically calculated from the patient’s weight and displayed as final numeric values on the AR headset of the medication nurse. In this TeamScreen-only prototype, dose-related panels (eg, tasks 8 and 16) primarily served to test whether participants could correctly locate where this information could be accessed rather than to verify the arithmetic itself. Similarly, the medication menu including succinylcholine (step 19) among other intubation drugs was configured to assess navigation and selection within the interface rather than as a recommendation for succinylcholine use during IHCA. An updated TeamScreen view reflecting the current implementation is provided in Multimedia Appendix 1.

Participants individually performed the 21 information retrieval tasks while progressing through a scripted walk-through simulated pediatric IHCA scenario, with clinical information and TeamScreen prototype screens presented in a structured, stepwise fashion. Each of the 20 participants completed the full scenario once, resulting in 20 standardized runs of the case.

**Table 1.** List of information retrieval tasks.

No.	Task name	Information retrieval task	Expected answer
1	Define weight I	Locate the weight entry area	Locate the weight entry area; no weight displayed^a^
2	Pulse check	Does the patient have a pulse?	Not indicated; it is in progress
3	Resuscitation time	How long has the resuscitation been going on?	15 s
4	Define weight II	What is the patient’s weight?	25 kg
5	CPR^b^ status	What is the status of CPR?	CPR has started; CPR was done once; CPR was performed for 10 s
6	Cardiac rhythm	What is the cardiac rhythm?	VF^c^; VF/pVT^d^
7	Shocks given	How many shocks were delivered?	1 (algorithm or completed tasks)
8	Medication status I	Can any medication be administered?	Yes (epinephrine)
9	CPR time	How much CPR time is left to do?	1 min
10	Secondary tasks	Which secondary task is proposed?	Prepare the second person for CPR
11	Next step	What is the next step?	Check the pulse; identify the cardiac rhythm; switch the CPR provider
12	A/B^e^ problem assessment	Which tasks have been carried out to resolve the A/B problems?	ETT^f^ intubation and repositioning
13	Intubation time	At what time did the intubation take place?	HH:MM^g^
14	Tasks in progress	Which tasks need to be done now?	Resume CPR for 2 min; administer epinephrine 0.01 mg/kg (0.1 mL/kg)
15	Shock time	At what time was the shock delivered?	HH:MM
16	Medication status II	Which medication was administered?	Epinephrine
17	Epinephrine time	How long until epinephrine must be administered again?	3 min 25 s
18	“Other tasks” section status	A team member placed an intraosseous line. Where is this information displayed?	Under “Completed Tasks”/“Other tasks”
19	Medication status	Which medication is being prepared?	Succinylcholine
20	Reversible causes	In the “Tasks in progress” area, the message “Treat the reversible causes” appeared. Where can you find help to recall the list of reversible causes?	Below the algorithm
21	Patient status: general understanding	What is the patient’s current condition?	No longer in cardiac arrest; moved to ROSC^h^; ABCDE^i^ assessment

a Success was defined as correctly identifying the relevant interface element rather than as giving a numeric answer.

b CPR: cardiopulmonary resuscitation.

c VF: ventricular fibrillation.

d pVT: pulseless ventricular tachycardia.

e A/B: airway and breathing.

f ETT: endotracheal tube.

g HH:MM: hours and minutes.

h ROSC: return of spontaneous circulation.

i ABCDE: airway, breathing, circulation, disability, exposure.

#### Outcome Measures

##### Quantitative Data

###### Performance Metrics

Participant performance was evaluated using 2 key efficiency metrics:

- Task success rate: tasks were classified as successful if the participant provided the expected answer (Table 1), either immediately or after a correction, as long as the final response was correct. Incorrect or incomplete responses were categorized as task failures.
- Time on task: This metric measured the average time (in seconds) taken to complete each task, starting from the end of the examiner’s instructions until the participant provided a final answer (Table 1). Since participants had no prior training with TeamScreen, response time was not used as a determinant of task success.

###### User Satisfaction

User satisfaction was measured after completion of the tasks using the PSSUQ version 3 [28], a 16-item instrument rated on a 7-point Likert scale (Multimedia Appendix 2), rated from 1 (“strongly agree”) to 7 (“strongly disagree”), in addition to a “not applicable” (N/A) option. Items 7 and 8, related to error messages and error recovery, were not applicable in this study because the TeamScreen prototype did not implement system error messaging or recovery workflows. These items were therefore marked “not applicable” and omitted from score calculations, in accordance with published PSSUQ scoring guidance [28,33], with overall scores computed as the mean of the remaining answered items.

The PSSUQ yields an overall usability score and scores on 3 subscales—system usefulness (items 1‐6), information quality (items 7‐12), and interface quality (items 13‐15)—with lower scores indicating greater satisfaction in each category. Based on 21 studies including 210 participants, the benchmark means reported by Lewis [34] provided the reference values for interpreting the PSSUQ scores: overall 2.82, system usefulness 2.80, information quality 3.02, and interface quality 2.49. This comparison with published benchmark values was purely descriptive. No inferential statistical testing was performed against these reference data. Better performance and satisfaction are reflected in lower PSSUQ scores (closer to 1).

### Qualitative Data

#### Error Analysis

All usability challenges encountered during task completion were systematically recorded and categorized using ergonomic criteria for human-computer interface evaluation [35]. Each usability issue was assigned a severity rating based on Nielsen Norman Group’s rating scale [36], assessing its impact on user performance and experience. Severity level 3 indicates a major usability problem that significantly hinders task execution, forcing users to find alternative solutions and requiring urgent corrective action. A severity level 2 represents a minor usability issue that may cause frustration but does not prevent task completion, warranting moderate priority for resolution. A severity level 1 is considered a cosmetic issue, affecting only aesthetics, with no impact on usability, requiring resolution only if time allows. Finally, a severity level of 0 is considered as not usability related. Findings from the severity assessment informed targeted design refinements for TeamScreen’s next iteration. The identified issues were addressed in a recently conducted multicenter simulation study, for which a manuscript is currently in preparation.

#### Think-Aloud Verbal Feedback

During testing, participants were encouraged to verbalize their thought processes while interacting with TeamScreen. These verbal reflections were analyzed to identify usability issues, inform content and design refinements, and improve system design for better real-world applicability.

#### Posttest Survey Feedback

To complement quantitative findings, qualitative feedback was collected via a standardized set of posttest interview questions (Table 2), ensuring consistency in data collection.

Responses provided a holistic understanding of user experience. Findings were systematically analyzed to refine TeamScreen’s interface and functionality, ensuring alignment with user expectations and clinical workflows.

**Table 2.** Posttest survey.

Question	Type of question	Choice provided
Display understanding		
1. What is your overall impression of the display?	Open-ended question	—^a^
2. What did you find easy to understand in this display?	Open-ended question	—
3. What did you find difficult to understand in this display?	Open-ended question	—
4. What do you think of the readability of the information (eg, font size, colors, etc)?	Open-ended question	—
5. If you had to rearrange the entire TeamScreen display, what ideas or suggestions for improvement would you propose?	Open-ended question	—
Completed tasks		
6. What difficulties did you encounter while using the display, and why?	Open-ended question	—
7. What aspects of the display helped you complete the tasks (information retrieval)?	Open-ended question	—
8. What aspects of the display hindered you from completing the tasks (information retrieval)?	Open-ended question	—
Display preferences		
9. What do you think of the distribution of information in the “Algorithm” section?	Open-ended question	—
10. What do you think of the distribution of information in the “Current Task” section?	Open-ended question	—
11. To what extent did you find the display of secondary tasks helpful regarding patient status?	Likert-scale question	Very useful Useful Neutral Somewhat useful Not useful at all
12. What do you think of the distribution of information in the “Next Steps” section?	Open-ended question	—
12a. How many “next steps” would you ideally want to see? One, two, three, or more?	Single-choice question	One step Two steps Three steps Other: ______
13. What do you think of the distribution of information in the “Countdown” section?	Open-ended question	—
14. What do you think of the distribution of information in the “Completed Tasks” section?	Open-ended question	—
15. What do you think of the distribution of information in the “Medications” section?	Open-ended question	—
15a. What do you think these three icons represent in the “Medications” section?	Closed-ended question	—

a Not applicable.

### Data Collection and Analysis

#### Data Collection and Confidentiality

To ensure data confidentiality, all collected materials, including recordings, online questionnaires, and consent documents, were coded. Recordings captured only participants’ screen view, eye movements, and voice, ensuring privacy. When verbatim quotes were used in qualitative data analysis or reporting, only the assigned identifier was displayed. Each usability testing session was video- and audio-recorded for retrospective analysis of TeamScreen’s usability. Two researchers (SO and ADM) independently reviewed task success rates and completion times. All PSSUQ scores were collected immediately after each test session and transcribed into Microsoft Excel spreadsheets for analysis. Half of the video recordings were independently assessed by SO and ADM to validate task success rates and completion times. Quantitative data collected during task execution underwent statistical analysis, and results were anonymized for reporting (see the *Results* section). During data collection and analysis, only 3 researchers (AR, SO, and ADM) had access to the audio and video recordings.

#### Data Analysis

Descriptive statistics were used to summarize continuous variables, including task success rates, time-on-task metrics, and PSSUQ scores. Frequency counts and percentages were used to analyze categorical variables, such as the types of usability errors. Additionally, a correlation analysis of task failure rates was performed to explore potential dependencies between tasks, indicating whether tasks shared common cognitive demands or interface elements. Quantitative data, including task durations and success rates, were analyzed using the *pandas* library in Python (version 3.12; Python Software Foundation) to identify trends and compute averages. Verbal feedback from the think-aloud sessions was audio-recorded, transcribed verbatim, and analyzed using a structured content analysis approach. Two researchers (AR and SO) independently reviewed the transcripts, segmented them into meaning units corresponding to distinct interface problems, and coded each unit using Bastien and Scapin’s [35] ergonomic criteria for human-computer interaction (guidance, workload, explicit Ccntrol, adaptability, error management, consistency, significance of codes, and compatibility). These criteria were chosen because they provide a well-established, task-oriented framework for classifying usability issues in interactive systems [37] and have been widely used in the evaluation of medical and health information technology interfaces [26,38]. Coded segments were then compared between both researchers. Discrepancies were resolved through discussion, and similar issues were grouped into overarching usability problems, which were mapped to specific interface sections (header, algorithm, completed tasks, medications). For each distinct usability issue, we calculated its frequency (number of participants who mentioned it) and assigned a severity rating using Nielsen Norman Group’s rating scale ranging from 0 to 4 (0=none, 4=catastrophic). Graphical representations were generated using *matplotlib*, an open-source Python library [39].

## Results

### Participant Characteristics

The usability tests were conducted in mid-September 2024. A total of 20 health care professionals participated in the study, of which 14 (70%) were women. The sample included 15 nurses (75%) and 5 physicians (25%). Of the 20 participants, 10 (50%) had completed PALS certification, including 3 of 5 physicians (60%) and 7 of 15 nurses (46.67%). Notably, 8 of 15 nurses (53.33%) had no PALS certification. The participants had a median experience of 6.00 (IQR 2.00-11.75) years in pediatric emergency care. All had prior exposure to real pediatric resuscitations, with the number of cases ranging from 2 to 100 and 30 cases being the most frequently reported value. Similarly, all had experience with simulated resuscitations, with the reported number of cases ranging widely, most commonly around 20 simulations.

Participants’ self-reported familiarity with digital devices varied: of 20 participants, 10 (50%) described themselves as “comfortable” using tablets and smartphones, whereas 6 (30%) reported being “very comfortable.” The remaining participants were either “neutral” (3/20, 15%) or “slightly uncomfortable” (1/20, 5%) with digital tools.

### Usability Evaluation

#### Quantitative Evaluation

##### Task Success Rate

**Figure 4.**
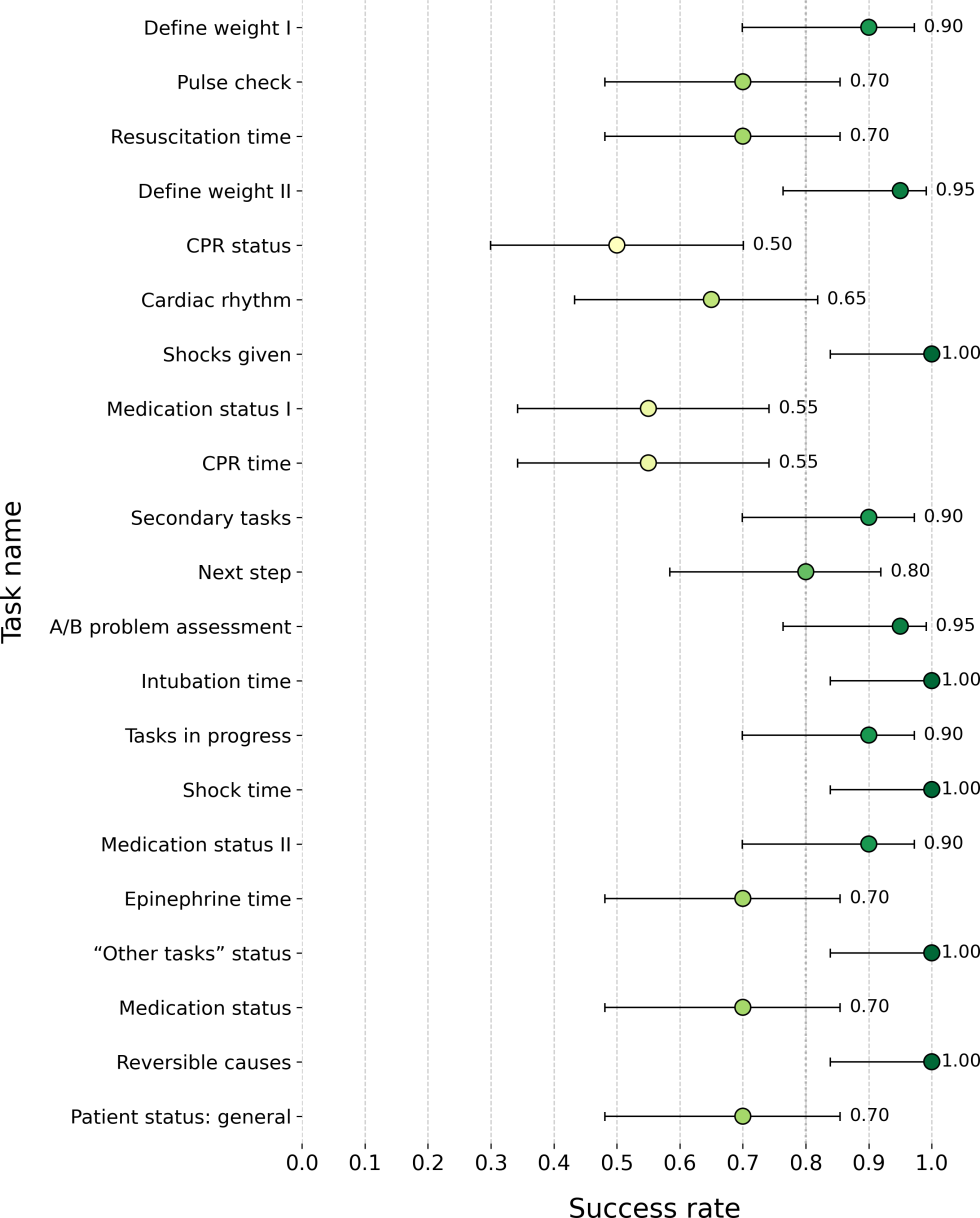
Mean (95% CI) success rate by information retrieval task. The color gradient indicates the task success rate, ranging from light yellow (lower success rates) to dark green (higher success rates). A/B, airway and breathing; CPR: cardiopulmonary resuscitation.

Task success rates varied significantly across the usability assessment (*χ*^2^_20_=74.59, *P*<.001; Figure 4), ranging from 50% to 100%, with an overall success rate of 81.19% (SD 16.87%). Highest success rates (100%) were achieved for tasks 7 (*Shocks given*), 13 (*Intubation time*), 15 (*Shock time*), 18 (*“Other tasks” section status*), and 20 (*Reversible causes*), indicating that they were consistently completed without errors. The lowest success rates were achieved for task 5 (*CPR status*; 50%), followed by tasks 8 and 9 (55% each), suggesting that these tasks were more cognitively demanding or required greater user adaptation.

##### Time on Task

**Figure 5.**
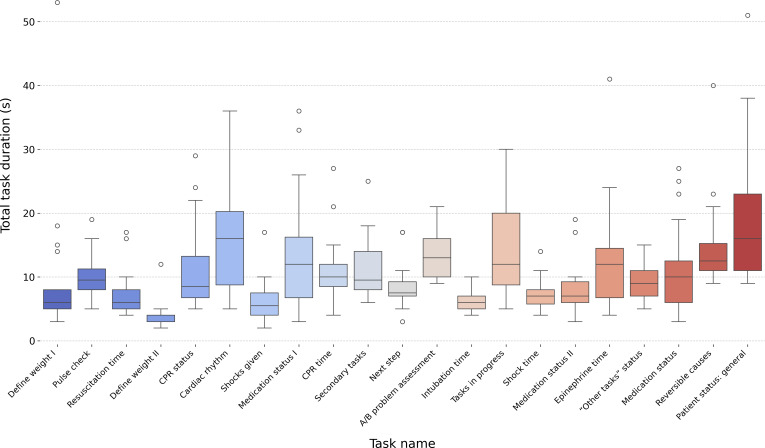
Box plot illustrating the distribution of task completion times across all 21 tasks performed during the usability study. Each box represents the IQR, with the median completion time indicated by the horizontal line inside the box. Whiskers extend to 1.5 times the IQR, and outliers are displayed as individual circles. Task 21 (Patient status: general understanding) exhibited the longest mean duration, suggesting higher complexity or additional steps required for completion, while task 4 (Define weight II) had the shortest duration, indicating a simpler information retrieval process. A/B: airway and breathing; CPR: cardiopulmonary resuscitation.

Task completion times varied significantly (Kruskal-Wallis H_20_=154.99, *P*<.001; Figure 5), reflecting differences in task complexity. On average, participants spent 170.80 (SD 44.70) seconds completing all 21 tasks, corresponding to a mean completion time of 8.13 (SD 7.07) seconds per task across all participants. Task 21 (*Patient status: general understanding*) required the longest duration, with an average completion time of 18.45 (SD 12.19) seconds, whereas task 4 (*Define weight II*) was completed the fastest, averaging 2.35 (SD 1.90) seconds.

##### Average Task Duration by Success Versus Failure

**Figure 6.**
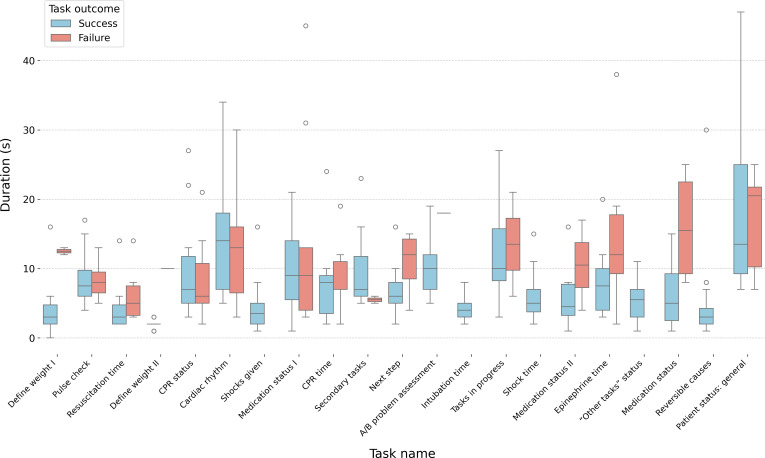
Box plot comparing task completion times between successful (blue) and failed (red) attempts across all 21 tasks. Each box represents the IQR, with the median duration indicated by the horizontal line. Whiskers extend to 1.5 times the IQR, and outliers are shown as individual circles. Overall, failed attempts generally took longer to complete, indicating challenges in task execution or repeated attempts (eg, task 1 [*Define weight I*]: failures averaged 12.5 s vs 3.77 s for successful attempts). However, for task 5 (*CPR status*), success and failure durations were nearly identical, suggesting that participants required similar effort regardless of the outcome. A/B: airway and breathing; CPR: cardiopulmonary resuscitation.

Analysis of task completion times based on success or failure revealed a notable trend (Figure 6). In most cases, failed attempts were associated with significantly longer durations, suggesting that unsuccessful users required multiple attempts or encountered execution challenges. For example, in task 1 (*Define weight I*), failed attempts averaged 12.50 (SD 0.71) seconds, whereas successful attempts took only 3.78 (SD 3.42) seconds (*P*=.048), highlighting the impact of task difficulty on completion time. However, in task 5 (*CPR status*), the completion times for successful attempts (mean 10.00, SD 8.25 seconds) and failed attempts (mean 8.30, SD 5.81 seconds) were nearly identical (*P*=.73), indicating that participants invested consistent effort regardless of outcome.

##### Correlation of Failure Rates Among Tasks

**Figure 7.**
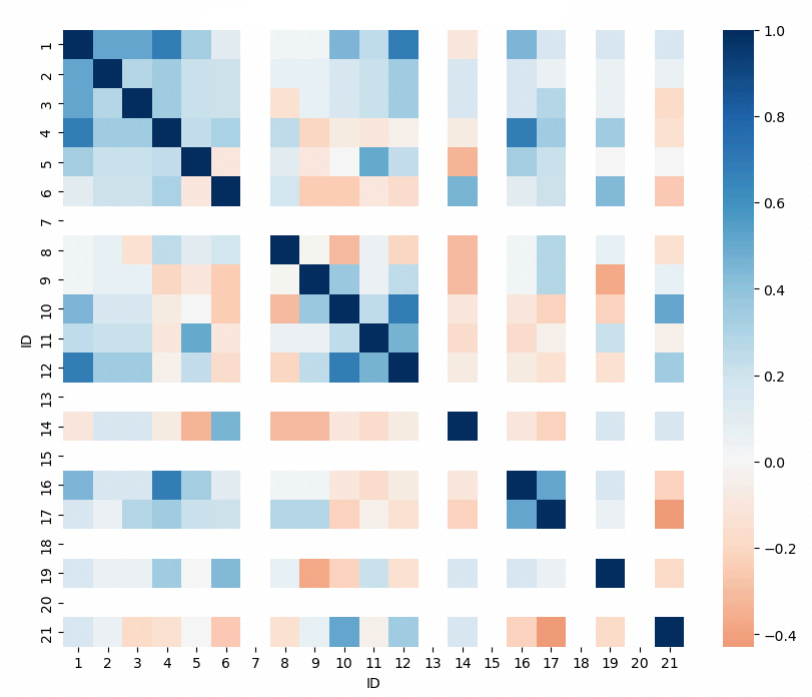
Heat map visualizing the correlation of failure rates across 21 usability tasks. Darker blue regions indicate strong positive correlations. Conversely, light red areas represent negative correlations, where failure in one task was less predictive of failure in another.

A correlation analysis of failure rates across tasks revealed potential dependencies, indicating that certain tasks share underlying cognitive demands or interface elements (Figure 7). Tasks with similar failure rates suggest common user difficulties, likely due to overlapping knowledge requirements or proximity within the interface. Several tasks exhibited strong positive correlations, suggesting that errors in one task were predictive of errors in another. For example, tasks 1 (*Define weight I*) and 4 (*Define weight II*) were highly correlated with tasks 12 (*A/B problem assessment*) and 16 (*Medication status II*) (Pearson correlation of per-participant binary success: *r*=0.69, *P*<.001), as all of these required users to track updated patient data, such as weight changes, completed airway and breathing interventions, and medication details. Likewise, task 4 (*Define weight II*) and task 16 (*Medication status II*) showed a strong correlation (*r*=0.69, *P*<.001), likely reflecting the dependency between understanding the patient’s weight and correctly administering weight-based medication. Task 10 (*Secondary tasks*) was significantly correlated with task 12 (*A/B problem assessment*; *r*=0.69, *P*<.001), suggesting that both tasks relied on the ability to monitor procedure logs or timeline view. Finally, tasks 16 (*Medication status II*) and 17 (*Epinephrine time*) exhibited a strong correlation (*r*=0.51, *P*=.02), as both involved understanding epinephrine administration; participants who struggled with one were likely to struggle with the other, highlighting potential cognitive or interface-related dependencies. Overall, these suggest that shared interface elements or related data points contributed to error clustering, emphasizing the need for enhanced visual cues or streamlined navigation to support information retrieval across interdependent tasks.

### Qualitative Evaluation

#### Frequent Errors

Analysis of task failures showed that task 5 (*CPR status*) had the highest failure frequency, accounting for 12.50% (10/80) of failed attempts. This was closely followed by tasks 8 (*Medication status I*) and 9 (*CPR time*) (9/80 each, 11.25%). Task 6 (*Cardiac rhythm*) accounted for 10% (8/80) of failures, and tasks 2, 3, 17, and 19 each accounted for 7.50% (6/80) of failures. These trends indicate that certain tasks posed consistent challenges across participants, potentially due to interface complexity, cognitive load, or unclear task presentation. Table 3 provides a detailed breakdown of failed task occurrences across the 21 tasks, considering 80 total opportunities across all tasks.

**Table 3.** Task failure frequency (N^a^=80).

Task number	Content	Failure frequency, n^b^ (%)
5	What is the status of CPR^c^?	10 (12.50)
8	Can any medication be administered?	9 (11.25)
9	How much CPR time is left to do?	9 (11.25)
6	What is the cardiac rhythm?	8 (10)
17	How long until epinephrine must be administered again?	6 (7.50)
2	Does the patient have a pulse?	6 (7.50)
19	Which medication is being prepared?	6 (7.50)
3	How long has the resuscitation been going on?	6 (7.50)
21	What is the patient’s current condition?	5 (6.25)
11	What is the next step?	4 (5)
16	Which medication was administered?	3 (3.75)
14	Which tasks need to be done now?	2 (2.50)
1	What is the patient’s weight?	2 (2.50)
10	Which secondary task is proposed to you?	2 (2.50)
12	Which tasks have been performed to address the A/B^d^ problems?	1 (1.25)
4	What is the patient’s weight?	1 (1.25)

a N: total number of opportunities.

b n: number of failed attempts.

c CPR: cardiopulmonary resuscitation.

d A/B: airway and breathing.

#### Encountered Usability Issues

A total of 16 usability issues were identified across different sections of the TeamScreen interface. These issues were analyzed using the heuristics of Bastien and Scapin [35] and classified by severity levels according to the Nielsen Norman Group’s rating scale [36] (Table 4).

**Table 4.** Classification of usability issues identified by participants (N=20).

Usability issues (description and section)	Frequency, n	Bastien and Scapin’s heuristics	Problem severity^a^
General issues			
*Excessive information displayed*: multiple panels (eg, algorithm, completed tasks, and medications) with many elements visible at once made it difficult to identify the most relevant information for the current resuscitation step (general screen layout).	10	*Workload*: brevity, information density	3
*Confusing time notation of CPR^b^ timer:* the CPR timer label did not clearly indicate whether the value represents total arrest duration, current cycle length, or time since last intervention, leading to misinterpretation of the timer.	2	*Significance of codes*	2
Header section			
*Location visibility of patient weight*: patient weight was located in the upper banner but not visually highlighted; several participants reported difficulty locating it quickly when asked to verify the weight.	5	*Guidance*: legibility	2
*Font size of patient weight*: the font size of patient weight in the header was too small to be read comfortably from the standard viewing distance of 2.7 m.	3	*Guidance*: legibility	1
*Lack of current time display*: the current clock time was not displayed anywhere on TeamScreen, forcing users to rely on external clocks to track absolute time during the scenario.	1	*Adaptability*	2
Algorithm section			
*Poor legend visibility*: the legend explaining the algorithm and color codes was small, making it difficult for users to remember or quickly decode the meaning of icons or colors.	4	*Guidance*: legibility	2
*Difficulty understanding transitions between algorithm branches*: transitions between algorithm branches (for example, from shockable to nonshockable pathways or post-ROSC^c^ care) caused uncertainty about which branch to follow next.	6	*Guidance*: prompting	3
*Visibility of patient status*: patient’s current state (eg, “cardiac arrest”) was not clearly highlighted near the active algorithm branch, which reduced situational awareness.	3	*Compatibility*	3
*Font size of 6H5T^d^ text*: the text listing reversible causes (6H5T) was presented in a small font and dense format, which participants found hard to read and use during the scenario.	3	*Guidance*: legibility	1
Completed tasks section			
*Overloaded with information*: the list of completed tasks showed all previous actions with detailed timestamps, resulting in a visually dense list that was difficult to scan for the most recent or most important actions.	5	*Workload*: brevity, information density	3
*Poor organization of task display in sections C^e^, A/B^f^*	3	*Compatibility*	2
*Insufficient separation between subsections*	3	*Guidance*: legibility	2
*Counterintuitive time notation*	3	*Significance of codes*	2
Medications section			
*Ambiguous icons*: icons used to represent medication-related actions (ie, in preparation, ready to administer, and administered) were not self-explanatory without accompanying text labels, potentially leading to misinterpretation of their meaning.	15	*Significance of codes*	3
*Missing calculated total doses/volumes*: the medications panel did not display the final calculated total dose or volume for each drug, requiring users to mentally calculate weight-based dosage, which increased cognitive load and potential for dosing error.	5	*Compatibility*	2
*Unclear listed medication doses*	3	*Compatibility*	2

a Nielsen Norman severity score: 4=catastrophic, 3=major, 2= minor, 1=cosmetic, 0=none.

b CPR: cardiopulmonary resuscitation.

c ROSC: return of spontaneous circulation.

d 6H5T: hypovolemia, hypoxia, hydrogen ion (acidosis), hypokalemia/hyperkalemia, hypoglycemia, hypothermia, tension pneumothorax, tamponade, toxins, thrombosis (pulmonary), thrombosis (coronary).

e C: circulation.

f A/B: airway and breathing.

The primary usability issues were general challenges rather than being confined to specific interface sections. These included cognitive overload due to excessive information displayed information; difficulty understanding task requirements, increasing mental workload; and medication-related confusion, particularly in interpreting drug administration icons. The most frequently reported issue was the lack of clarity in medication icons, making it difficult for participants to distinguish between medications in preparation, ready for administration, or already administered. Other issues, although present, were encountered by fewer than one-third of participants.

Overall, the study identified key usability challenges related to information visibility, navigation, and comprehension. The most critical usability issues (severity 3‐4) included medication icon confusion (most frequently reported), excessive information density leading to cognitive overload.

#### User Satisfaction

##### PSSUQ Scores

Participants provided positive feedback on screen readability, with a mean PSSUQ score of 2.40 (SD 1.24) indicating high usability. However, they suggested improvements in information hierarchy and task simplification due to perceived cognitive overload. Overall, PSSUQ scores were lower (better usability) than the PSSUQ version 3 benchmark (Table 5), confirming high system usefulness and interface quality.

Scores closer to 1 indicate higher usability. TeamScreen scores were descriptively lower than the published PSSUQ benchmark means, suggesting that participants found the system intuitive and effective, although information presentation still offers room for optimization.

**Table 5.** Comparison of TeamScreen PSSUQ^a^ scores versus the PSSUQ version 3 (V3) benchmark.

	TeamScreen score, mean (SD)	PSSUQ V3 benchmark mean
Overall score	2.40 (1.24)	2.82
System usefulness (SysUse)	2.44 (1.22)	2.80
Information quality (InfoQual)	2.41 (1.20)	3.02
Interface quality (IntQual)	2.20 (1.41)	2.49

a PSSUQ: Post-Study System Usability Questionnaire.

##### Participant Verbal Feedback

Think-aloud verbalizations during task execution, combined with responses from the posttask debriefing survey, provided valuable contextual information that enriched the interpretation of the PSSUQ scores and offered deeper insights into users’ experiences and expectations regarding the interface.

Overall, participants expressed mixed feelings about their experience with the TeamScreen interface. In terms of strengths, 8 of 20 (40%) participants highlighted the clarity and organization of the information, quality of the display, and good readability (in terms of font size and color). Several participants particularly appreciated the guidance provided through the algorithm section (12/20, 60%) and the associated color coding (8/20, 40%)—green indicating “finished” and orange indicating “in progress”—as well as the “Completed Tasks” section (9/20, 45%), which enhanced their situational awareness.

Regarding areas for improvement, 9 of 20 (45%) participants reported misunderstanding difficulties, particularly related to the middle section of the screen, not directly attributable to usability issues. Several users (10/20, 50%) reported that the amount of information presented was overwhelming, contributing to increased cognitive load. The “Current Task” area was also frequently overlooked, as 9 of 20 (45%) participants felt that the relevant information was already accessible within the algorithm panel on the left side of the screen, making it redundant. Furthermore, several users suggested making a clearer distinction between “Current Task” and “Next Steps” to better support cognitive processing under pressure. These insights emphasize the need for improvements in information hierarchy and cognitive load reduction in critical usage scenarios. Finally, participants indicated a need for greater clarity in the “Medication section” (5/20, 25%), specifically concerning the meaning of the 3 icons representing the medication status (in preparation, ready for administration, administered). However, it is important to acknowledge that many of these reported issues were, according to the participants, largely attributable to their lack of prior exposure to the app. Participants indicated that with appropriate training, the interface would become significantly more intuitive, enabling faster and more accurate information retrieval during real-time pediatric cardiac arrest scenarios, as well as more effective adherence to American Heart Association guidelines.

### Modifications Based on Usability Findings

Following the usability analysis, several targeted refinements were implemented, each addressing a specific usability issue summarized in Table 4. First, limited visibility and ambiguous interpretation of the CPR timer led us to relocate the main timer to the top of the screen and clarify its label to ensure continuous and unambiguous visibility throughout the resuscitation process. Second, difficulties in reading and using the reversible causes (6H5T; hypovolemia, hypoxia, hydrogen ion [acidosis], hypokalemia/hyperkalemia, hypoglycemia, hypothermia, tension pneumothorax, tamponade, toxins, thrombosis [pulmonary], thrombosis [coronary]) prompted an increase in font size and a permanent display to provide immediate access to correction measures. Third, issues related to information overload and poor readability of the list of completed tasks motivated a restyling of the task list, replacing square markers with bullet points, and secondary tasks were repositioned higher in the interface to make them more noticeable and actionable. Fourth, in response to concerns that timers were easy to overlook as they approached zero, countdown timers were enhanced to blink when nearing expiry, drawing the team’s attention and reducing the risk of delayed interventions. Finally, frequent misinterpretation of medication icons and confusion around drug administration stages led to the introduction of 3 distinct, clearly labeled icons indicating whether a drug was in preparation, prepared, or already administered. Collectively, these modifications aimed to alleviate information overload, improve navigation, and highlight essential cues in high‐stress pediatric resuscitation scenarios.

## Discussion

### Key Findings

TeamScreen introduces a new generation of context-aware clinical displays aimed at reinforcing shared cognition and temporal coordination among resuscitation teams rather than replacing clinical judgment. This single-center, mixed methods study evaluating its usability during simulated IHCA management yielded several salient insights.

With an overall task success rate of 81%, most participants effectively located and interpreted essential clinical information under simulated urgency, despite challenges in early pediatric IHCA response [40]. Tasks 7 (*Shocks given*), 13 (*Intubation time*), 15 (*Shock time*), 18 (*“Other tasks” section status*), and 20 (*Reversible causes*), covering shock delivery logs, intubation timestamps, and reversible‐causes guidance, achieved 100% success, suggesting that these interface elements were well designed and intuitive. Conversely, tasks 5 (*CPR status*), 8 (*Medication status I*), and 9 (*CPR time*) related to tracking ongoing CPR and upcoming interventions (eg, epinephrine administration) exhibited the highest failure rates. Participants who struggled with these tasks frequently cited uncertainty regarding the location of real-time information, highlighting a need for improved visual hierarchy in high-density interface areas, in line with cognitive load theory [41].

Task completion times demonstrated substantial variability across the 21 tasks, reflecting heterogeneity in task complexity and cognitive processing demands. Task 21 (*Patient status: general understanding*) exhibited the longest mean completion time, indicating that tasks involving a broader synthesis of patient information were more time-consuming. Conversely, task 4 (*Define weight II*) was completed most rapidly, suggesting minimal cognitive load and straightforward retrieval. This efficiency may also be attributed to task 4 representing the second occurrence of a previous task, that is, task 1 (*Define weight I*), whereby participants were already familiar with the location of the relevant information on the display.

Correlation analysis showed errors clustering around related data points, such as medication timing and dosage, underscoring the importance of consistent navigation cues and harmonized data presentation across the algorithm view, timeline, and “Completed Tasks” sections. This aligns with evidence that protocol adherence significantly improves IHCA outcomes [42], emphasizing TeamScreen’s potential to enhance care quality through better design. The PSSUQ scores indicated high user satisfaction, with participants valuing TeamScreen’s consolidation of vital parameters, current tasks, and next‐step recommendations into a central, readable display. These findings suggest that TeamScreen excels where clarity is prioritized (eg, timestamps and shock logs) but requires refinement in areas with visual clutter, icon ambiguity, or subtle task distinctions. Given varying roles in IHCA response where nurses often manage documentation and physicians lead decisions [43], future studies should explore whether task success or perceived usability differs across these groups.

### Comparison With Literature

TeamScreen shares similarities with real-time dashboard systems aggregating patient vitals, medication records, and intervention timelines onto shared displays, as seen in prior work improving clinicians’ access to critical data and reducing cognitive load [44]. However, unlike passive aggregators, TeamScreen integrates real-time visualization with interactive PALS-aligned algorithmic guidance, supporting both team situational awareness and decision-making during IHCA. This dual functionality addresses a gap in traditional dashboards, which often lack structured support for resuscitation guidelines. For instance, Siebert et al [45] demonstrated that AR glasses improved defibrillation dosing adherence compared to PALS pocket cards, although without broader workflow integration. A recent systematic review by Cheng et al [46] highlights the potential of AR and virtual reality to enhance resuscitation skills but notes inconsistent superiority over traditional methods, underscoring the need for integrated solutions such as TeamScreen. TeamScreen extends this concept by offering a centralized, algorithm-synchronized interface, positioning it within an emerging class of digital tools designed to optimize shared cognition and temporal alignment in high-acuity care.

### Essential TeamScreen Enhancements

The usability evaluation of TeamScreen has identified critical areas for future refinement, and the following enhancements will be implemented to optimize its support for resuscitation teams. The information hierarchy will be restructured to minimize visual overload, consolidating related elements such as medication timing and dosage into a distinct, clearly labeled panel, guided by cognitive load principles [41]. This adjustment could streamline data retrieval and lessen cognitive strain during time-sensitive tasks. Dynamic visual cues, such as color shifts or blinking countdowns for medication intervals, could prompt timely actions, provided they adhere to ergonomic principles to avoid distraction in high‐stress settings. Functionally, adaptive role-based prompts tied to PALS algorithms could guide users step-by-step, improving coordination and adherence. Standardizing labels, icons, and visual styles (eg, font sizes, colors, and legends) would enhance consistency and reduce the likelihood of misinterpretation, particularly for the 6H5T mnemonic and streamlined task lists. Finally, given its intuitive design, brief, role‐specific onboarding is advised—especially for users with varying digital fluency—to ensure efficient navigation in multitasking, high-stakes scenarios such as CPR. Paired with these design refinements and enhancements, targeted training could maximize TeamScreen’s potential to support resuscitation teams effectively.

### Study Limitations

This study has several limitations. Testing TeamScreen in a simulated environment without direct user interaction limits conclusions about real-world PALS algorithms adherence or patient outcomes. The heightened stress of a real-life IHCA situation might exacerbate usability challenges not fully captured in this study. Additionally, evaluating TeamScreen isolation (ie, without AR Headsets or Guiding Pad) potentially reduces ecological validity, as the full InterFACE-AR suite’s interplay was not assessed. This may also not fully capture the usability of the complete system. However, the Guiding Pad and AR headset modules have been evaluated in separate component-level usability studies, and the integrated InterFACE-AR system has recently been tested in a multicenter randomized controlled trial in a high-fidelity simulated setting. Third, the passive display design, reliant on a charting nurse’s input via the Guiding Pad, aligns with its intended use but restricted participants’ ability to explore the interface freely, potentially skewing engagement. Fourth, the study sample size and various clinical backgrounds of participants further limit generalizability. Fifth, our evaluation was supposedly conducted in a dedicated emergency department resuscitation bay using a fixed, wall-mounted TeamScreen display, which may limit generalizability to ward-based pediatric IHCA where mobile configurations of TeamScreen and the Guiding Pad would be required and should be evaluated in future work. Finally, while cognitive overload was frequently noted in feedback, this dimension remained unquantified owing to the absence of a validated instrument such as the NASA-Task Load Index (NASA-TLX) [47]. This should be addressed in future studies.

### Future Directions

Several of the usability issues identified in this study point to general design principles that are relevant for other digital tools in acute care. Information that is crucial for safe care (such as timers, current patient status, and key tasks) should be clearly visible on a shared team display. The amount of information on screen needs to be limited and well structured to avoid overload, and icons or color codes must be immediately understandable, even under stress. These lessons can guide the design of other real-time decision support systems that aim to support teamwork rather than only individual users.

The next phase of our program consists of 2 multicenter, simulation randomized controlled trials in 2025. The first, conducted in the second quarter of 2025, has now been completed, and the second, initiated in the fourth quarter of 2025, is currently underway, integrating TeamScreen with AR Headsets and Guiding Pad to evaluate clinical decision‐making, teamwork, communication, and patient safety outcomes across diverse hospital settings. Structured, role-specific onboarding will mitigate the learning curve effects observed in this study, offering a clearer performance baseline under realistic pressures. Because adherence to advanced life support guidelines is associated with better IHCA outcomes [42], metrics such as drug administration timing, error rates, and PALS adherence will provide deeper insights into how digital support influences care quality. Role differences (eg, nurses’ documentation vs physicians’ leadership [43]) will also be examined to further adapt the interfaces. Cognitive workload will be quantified using tools such as the NASA-TLX [47] to address qualitative feedback about overload and to test whether the system genuinely reduces cognitive burden.

Future iterations could incorporate artificial intelligence–driven predictive analytics to anticipate tasks (eg, rhythm reassessment), further reducing manual tracking demands. Qualitative feedback and high usability scores suggest that TeamScreen may alleviate cognitive load, although formal workload assessments are needed. Future work also needs to address more practical questions that apply to many medical digital tools, including feasibility of deployment, interoperability with existing hospital systems, and implementation costs, for widespread adoption. Integration with electronic health records, automated monitoring data capture, and tailored role-specific interfaces represent key translational goals to support clinical use of InterFACE-AR and similar decision support systems.

### Conclusion

This study provides a structured usability evaluation of TeamScreen, a shared digital interface designed to assist pediatric resuscitation teams during IHCA, with its clinical impact still to be validated. Using a mixed methods approach, we identified its strengths and limitations in a simulated setting. Results revealed an 81% task success rate and high user satisfaction, confirming TeamScreen’s ability to deliver accessible, interpretable clinical data under time pressure. However, issues with information hierarchy and task clarity, evidenced by correlated failures in navigation and updates, highlighted areas for refinement. The combined analyses of error categorization, verbal feedback, and questionnaire responses provided insights about the prototype’s strengths and areas requiring refinement. This comprehensive evaluation not only identified critical usability challenges but also validated the potential of the TeamScreen prototype to optimize pediatric resuscitation workflows.

## Supplementary material

10.2196/78736 Multimedia Appendix 1 Updated TeamScreen prototype display during a simulated pediatric in-hospital cardiac arrest (Ventricular Fibrillation scenario), showing the cardiopulmonary resuscitation (CPR) algorithm and reversible causes (left); timers and task lists for current, secondary and next actions (center); and the “Completed Tasks” panel with a medications table that presents the final weight-based doses and administration times visible to the entire team.

10.2196/78736 Multimedia Appendix 2 Post-Study System Usability Questionnaire (PSSUQ) statements.

## Abbreviations

6H5T: hypovolemia, hypoxia, hydrogen ion (acidosis), hypokalemia/hyperkalemia, hypoglycemia, hypothermia, tension pneumothorax, tamponade, toxins, thrombosis (pulmonary), thrombosis (coronary)
AR: augmented reality
CPR: cardiopulmonary resuscitation
IHCA: in-hospital cardiac arrest
InterFACE: Interconnected and Focused Mobile Applications on Patient Care Environment
PALS: pediatric advanced life support
PSSUQ: Post-Study System Usability Questionnaire
ROSC: return of spontaneous circulation
TLX: Task Load Index
