# Supplementary figures and images for: Evaluating a Shared Decision Support Tool for Pediatric Cardiopulmonary Arrest: Mixed Methods Usability Study
Source: JMIR Hum Factors. 2026 Apr 28;13:e78736. doi: 10.2196/78736 (PMC13123637; doi:10.2196/78736)

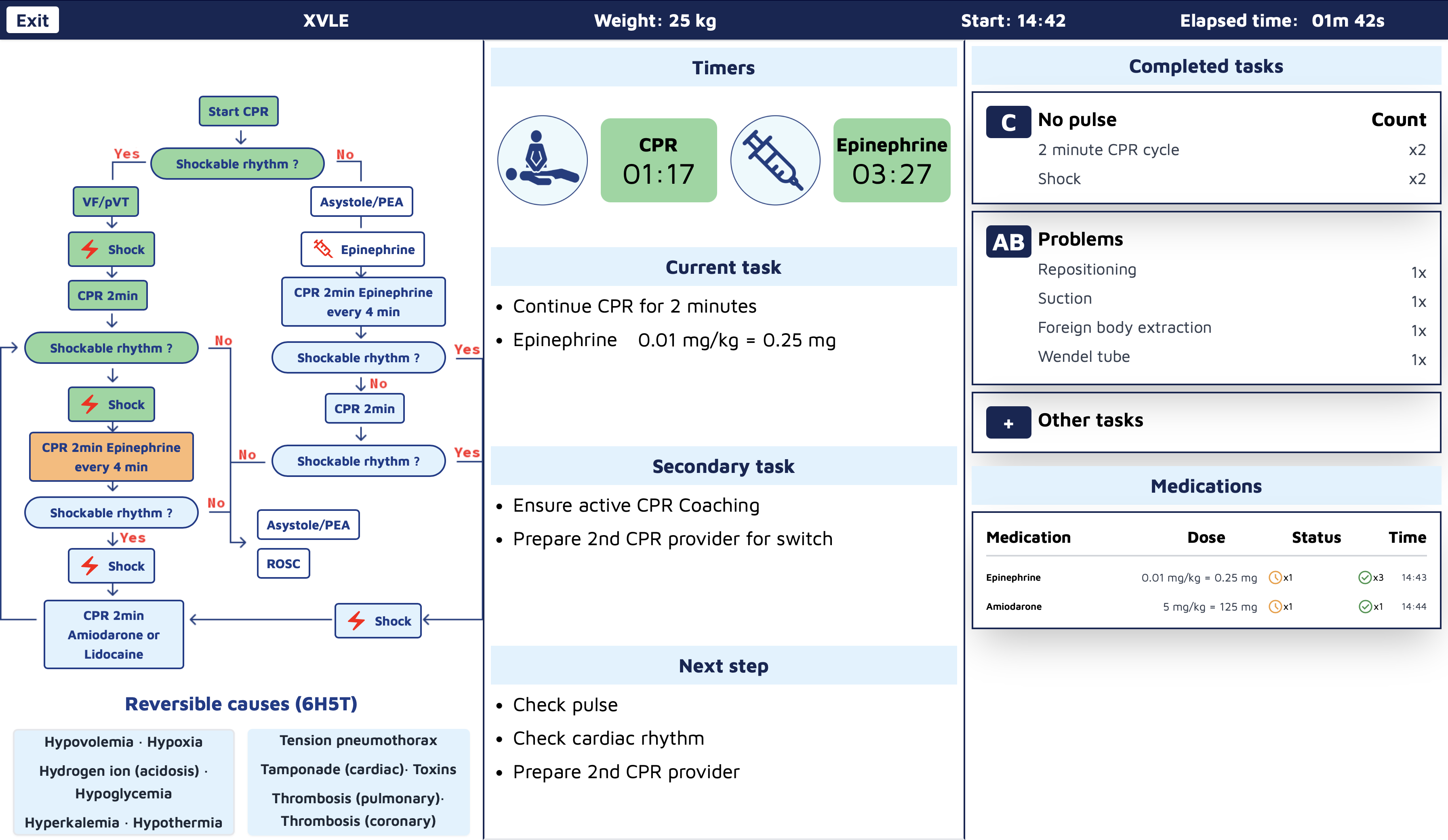

Supplement: Multimedia Appendix 1 [file humanfactors-v13-e78736-s001.png]
