# Supplementary material for: Evaluating a Shared Decision Support Tool for Pediatric Cardiopulmonary Arrest: Mixed Methods Usability Study
Source: JMIR Hum Factors. 2026 Apr 28;13:e78736. doi: 10.2196/78736 (PMC13123637; doi:10.2196/78736)
Supplement: Multimedia Appendix 2 [file humanfactors-v13-e78736-s002.docx]

Table S1. Post-Study System Usability Questionnaire (PSSUQ) statements.

| **Statement** | **Rating^a^** | | | | | | | |  |
| --- | --- | --- | --- | --- | --- | --- | --- | --- | --- |
| Overall, I am satisfied with how easy it is to use this system. | 1 | 2 | 3 | 4 | 5 | 6 | 7 | N/A | |
| It was simple to use this system. | 1 | 2 | 3 | 4 | 5 | 6 | 7 | N/A | |
| I was able to complete the tasks and scenarios quickly using this system. | 1 | 2 | 3 | 4 | 5 | 6 | 7 | N/A | |
| I felt comfortable using this system. | 1 | 2 | 3 | 4 | 5 | 6 | 7 | N/A | |
| It was easy to learn to use this system. | 1 | 2 | 3 | 4 | 5 | 6 | 7 | N/A | |
| I believe I could become productive quickly using this system. | 1 | 2 | 3 | 4 | 5 | 6 | 7 | N/A | |
| The system gave error messages that clearly told me how to fix problems. | 1 | 2 | 3 | 4 | 5 | 6 | 7 | N/A | |
| Whenever I made a mistake using the system, I could recover easily and quickly. | 1 | 2 | 3 | 4 | 5 | 6 | 7 | N/A | |
| The information (such as online help, on-screen messages, and other documentation) provided with this system was clear. | 1 | 2 | 3 | 4 | 5 | 6 | 7 | N/A | |
| It was easy to find the information I needed. | 1 | 2 | 3 | 4 | 5 | 6 | 7 | N/A | |
| The information was effective in helping me complete the tasks and scenarios. | 1 | 2 | 3 | 4 | 5 | 6 | 7 | N/A | |
| The organization of information on the system screens was clear. | 1 | 2 | 3 | 4 | 5 | 6 | 7 | N/A | |
| The interface of this system was pleasant. | 1 | 2 | 3 | 4 | 5 | 6 | 7 | N/A | |
| I liked using the interface of this system. | 1 | 2 | 3 | 4 | 5 | 6 | 7 | N/A | |
| This system has all the functions and capabilities I expect it to have. | 1 | 2 | 3 | 4 | 5 | 6 | 7 | N/A | |
| Overall, I am satisfied with this system. | 1 | 2 | 3 | 4 | 5 | 6 | 7 | N/A | |

^a^Ratings were assigned on a 7-point Likert scale, ranging from 1 (“strongly agree”) to 7 (“strongly disagree”), in addition to a “not applicable” (N/A) option.
